# Earliest evidence for systematic use of ultrahigh carbon steel in the ancient Aegean in the Archaic Milesia

**DOI:** 10.1371/journal.pone.0312244

**Published:** 2025-03-13

**Authors:** Ümit Güder, Jana Mokrišová, Marek Verčík, Ünsal Yalçın

**Affiliations:** 1 Institute of Classical Archaeology, Charles University, Prague, Czech Republic; 2 Max Planck Institute for Sustainable Materials, Düsseldorf, Germany; 3 Faculty of Classics, University of Cambridge, Cambridge, United Kingdom; 4 Deutsches Bergbau Museum, Bochum, Germany; 5 Süleyman Demirel University, Isparta, Türkiye; German Archaeological Institute: Deutsches Archaologisches Institut, GERMANY

## Abstract

This study presents the results of archaeometallurgical investigation of iron objects from the Sanctuary of Apollo in ancient Didyma, dating to the Archaic period (7^th^ to the early 5^th^ centuries BCE). The analysed precision work tools and semi-formed objects exhibit distinct material characteristics that differentiate them from other iron-steel artefacts of both small and large formats (weapons, implements, and architectural fittings) so far investigated in the Aegean. They were made of medium, high, and ultra-high carbon steel. Three objects belonging to this latter category consist of remarkably clean, homogeneous, and high-quality steel. After presenting the analytical results, this article discusses various explanatory models for the production of these objects and attempts to answer questions about the motivations for this innovation, highlighting their functional characteristics and context of production and consumption.

## 1. Introduction

**Fig 1:**
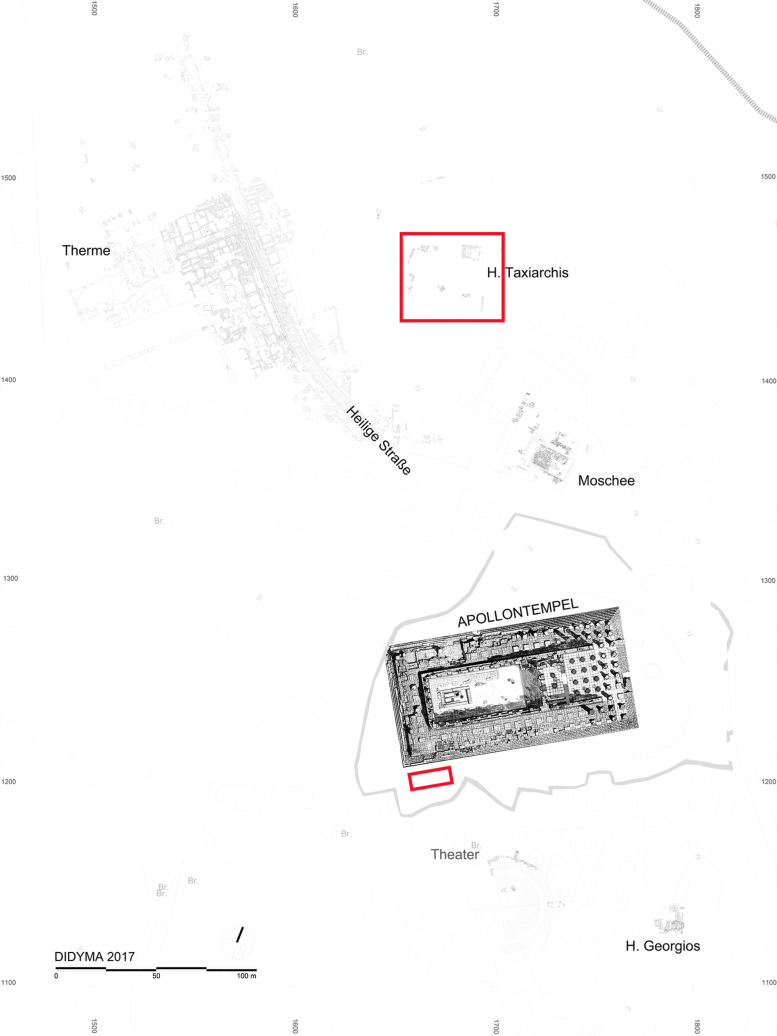
Plan of the Sanctuary of Apollo in Didyma. Findspots of objects discussed in the text are marked by red rectangles (based on the topographical survey of D. Hoffmann in 1997, H. Birk in 2009-2013, J. Goischke in 2013-2015, J. Falkenberg in 2016, and G. Fellner in 2017; digitisation of architectural surveys/ CAD by M. Saleh in 2016-2017 and M. Kohnert in 2017).

This paper presents archaeometallurgical examination of iron objects from the Sanctuary of Apollo in ancient Didyma dating to the Archaic period (7^th^ to the early 5^th^ centuries BCE). The oracular sanctuary is situated on the northwestern shores of the Gulf of Güllük (ancient Gulf of Iasos), located in the municipality of Didim, the prefecture of Aydin in western Türkiye (Fig 1; COORD: 37°23′06″N 27°15′23″E).

Analytical work identified an assemblage of precision work tools and semi-formed objects manufactured from ultra-high carbon steel (UHCS). This material has so far been documented in the tools of the Roman period [1], with the exception of previous investigations by Ü. Yalçın [2,3], who identified a single finished artefact made with UHCS at nearby Miletus, a major metropolis of southern Ionia. This study significantly expands and builds upon this isolated find and presents the earliest evidence for a systematic use of UHCS in the ancient Aegean yet. It explains the presence of such artefacts as a ‘test group’ used to drive metallurgical innovation, applied to a specific class of objects at a specified time and place.

In what follows we introduce the archaeological context and research focus, followed by a detailed analysis of microstructural and compositional characteristics of selected analysed objects. We address the lack of comparable data from both the Aegean and the eastern Mediterranean and provide a critical analysis of established explanatory models of processes applied during production. Furthermore, the function and use of these objects is discussed with respect to the distinct context of their deposition in the sanctuary at Didyma, the supra-regional significance of which elucidates the motivations behind the utilisation of the UHCS in this particular context. Finally, by taking technological, archaeological, and socioeconomic aspects of the assemblage into consideration, we consider innovations and discrete technological strands in iron working in the Archaic Aegean.

### 1.1. Archaeological setting

The extra-urban sanctuary at Didyma was one of the central places of worship for nearby Miletus, which was connected to it by the Sacred Road [4]. Established or (re)founded in the Late Geometric period (second half of the 8^th^ century BCE), the oracle became known far beyond the Aegean during the 7^th^ and 6^th^ centuries BCE [5]. The activity here abruptly came to an end during the Ionian revolt, when the Temple of Apollo was looted and destroyed by the Persians in 494 BCE. The decline was of a temporary duration only, as the construction of a new temple and the revival of the oracle began in the second half of the 4^th^ century BCE [6].

Recent excavations directed by Prof. Helga Bumke in 2000-2009 uncovered a largely undisturbed archaeological context on the Taxiarchis hill, located some 200 meters north of the Temple of Apollo. As reported by Bumke [5], the deposits consisted of five main stratigraphic levels. The oldest cultural level (“Ältester Kulturhorizont“) is located directly on the bedrock and contains disposed Late Geometric and Archaic votives. In the late 6^th^ or early 5^th^ century BCE, two debris levels (“Splittschicht” and “lehmiges Erdpacket”) were deposited here, probably in relation to construction work in the temenos, followed by a massive ashy level (“Kohlenhaltige Schicht”) of the early 5^th^ century BCE. Based on the finds’ composition and stratigraphic record (i.e., overlying sealing), the ashy level has been interpreted as the remains of the Persian destruction of the sanctuary, which were cleared from a wider area and moved to the Taxiarchis hill sometime after 494 BCE. The ashy level was overlaid by an early 5^th^ century BCE limestone sealing (“Kalksteinblokage”) and late during the Hellenistic period by a limestone chip layer (“nacharchaische Kalksplittschicht”) containing both archaic and post-archaic findings [5].

Noteworthy feature of the Archaic levels is the high number of excavated iron objects. The quantity and overall quality of the objects allow a further characterisation of the archaeological context. While the lowermost three Late Archaic levels contain for the most part dedications, cultic and ritual objects (e.g., weapons, obeloi), a considerable number of items of practical use (e.g., tools and implements, architectural fittings) has been found in the upper ashy level associated with the destruction [7]. Among this ‘rubbish’, a notable number of objects connected with craftspeople’s activities stands out [8]: iron slags (smithing hearth bottom (SHB) or plano-convex bottom (PCB) slags and amorphous pieces), cupriferous slags (amorphous and drops), semi-formed objects and billets, as well as metallurgical waste. They suggest that complex metallurgical operations were conducted at the site: blacksmithing, copper-alloy working, as well as lead and potentially also precious metallurgy [7,9].

### 1.2. Research background and theoretical framework

The Ancient Aegean experienced striking cultural, economic, artistic, and intellectual developments during the Archaic period (700-480 BCE) [10–14]. Literary evidence suggests that iron working played a significant role within this flourishing innovative environment [15,16]. The recorded achievements in the working of iron are best represented by Glaucus of Chios, who, according to Herodotus (I.25; writing in the mid-5^th^ century BCE), invented forge welding when he made a great silver bowl on an iron stand for the Lydian king Alyattes (ca 640-580 BCE). The depictions on late Archaic Attic (ca 500 BCE) black and red figure vases display in detail various tools and installations used by blacksmiths [17,18], but reveal very little about the techniques employed and knowledge available to craftspeople. Epigraphic evidence related to metallurgical, or metalworking practices is not of much help here either, as the first mentions of these processes date to the Hellenistic period [16].

**Fig 2:**
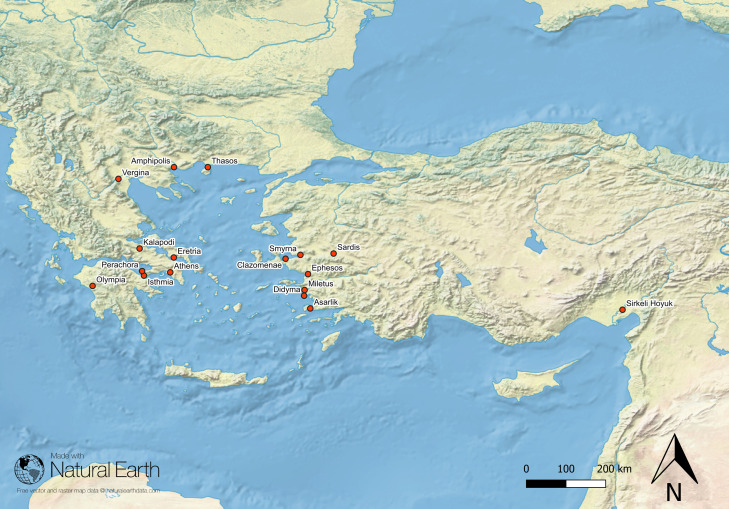
Map showing ancient sites mentioned in the text. Map by M. Šmolková (based on free vector and raster map from Natural Earth constructed with Qgis Software Version 3.4.11).

A brief glance at the present state of research reveals that our knowledge of Archaic iron technology is still elusive. Archaeological studies of iron in the Archaic Aegean (Fig 2) are generally strongly affected by the very poor state of preservation. As a result, they have often focused on typological, chronological, and chorographic investigations of objects, including those related to metallurgical operations, and relied on archaeological indicia such as tools, semi-formed objects, and bars [19–22]. Metallurgical remains have traditionally been regarded as the only direct proxies for iron production within settlements and sanctuaries [23–26]. And while slags have been frequently subjected to chemical characterisation in order to determine their type and origins [2,27–32], only a few analytical datasets that inform on microstructure and chemical composition of manufactured iron objects are available. Although these can be complemented by a few additional datasets from the Classical period, the interpretation of the Archaic data remains difficult. This is principally due to three different factors. The first is that the study of analysed artefacts has been either divorced from or associated only in a limited way with the archaeological context [33–39]. The second is that selected artefacts are dedications and offerings, comprising a specific category of finds not indicative of the local production [34,40]. The third is that these artefacts are often linked to very specific and singular contexts without a clear relationship to production sites (e.g., dedications at sanctuary coming from elsewhere), with little significance for a broader understanding of local skills and techniques applied [41]. Consequently, previous reconstructions have preferred a linear development of iron technology from the Early Iron Age onwards, envisioning a series of rather simple or a few advanced, techniques mastered by blacksmiths without closely considering diachronically the specifics of local traditions and knowledge-scapes [42–44].

Recent regional studies have started to shed the necessary light not only on the debates surrounding the introduction of iron [45,46], but also the innovations and technological strands concerning metals in the Archaic period [47–49]. For example, theoretically driven approaches combined with contextualised cutting edge analytical methods have been applied recently to test different models of technological (ex)change, paying attention to the varying sociocultural and environmental circumstances in the northern Aegean [50–52]. A similar archaeological and analytical program was initiated by the authors in 2017 with the aim to study iron from Early Iron Age and Archaic settlements and cult sites from ancient Ionia. The metallurgy in the region was not a significant focus of investigations until the late 1990s, with the single exception of the previously mentioned small assemblage of seven objects from Miletus analysed by Ü. Yalçın [2,3]. Iron from Sardis [53,54] comes, strictly speaking, from the neighbouring region of Lydia, but the published analytical data are of particular significance because of the attested interaction between the western and central part of Anatolia during the Archaic period. This picture has changed in the last decade and our dataset has been crucially extended by finds from the Gulf of Izmir, the Milesia, and Samos [55–60]. This allows us to investigate different technological requirements and choices implemented during iron working, which can then be studied with respect to local sociocultural and environmental circumstances.

The outlined stratigraphic, formal, and functional framework of the iron finds excavated at the Taxiarchis hill in ancient Didyma is essential for tracing regional technological developments during the Archaic period for two reasons. First is that these iron objects were utilitarian and local, used and produced at the site, or at least in its vicinity. Second is that the destruction of 494 BCE represents a unique well-defined terminus *ante quem* in a geographically defined region, the Milesian peninsula, a significant area in south Ionia. The combination of these conditions allows an application of the principle of ‘reverse engineering’ to the processes involved in the making of the objects in order to identify possible points of experimentation in production processes as well as to reconstruct a possible implementation of advanced smithing techniques. This in turn enables an evaluation of the technological and formal evolution of iron tools and implements that were deposited here by the late Archaic horizon. This approach cannot be applied to the well-studied weapons, decorative elements, and vessels that were dedicated in Archaic sanctuaries in great numbers [21,61], as they are usually not produced locally and barely have a functional purpose.

The ‘reverse engineering’ approach has been successfully applied to study innovation in bronze metallurgy [62]. Of specific relevance are case studies from the Archaic period suggesting that a selected group of objects might have served as a ‘test group’ for experimentation by ancient craftspeople [14]: for example, the search for bigger formats of highly prestigious cauldrons with three supporting legs (tripods) led to an increased ability to smelt copper-alloys and acted as a platform for experimentation with casting of fine ornaments. The reason behind implementing a test group of objects is that it is easier to control an experiment if it is limited to a few types -- rather than a whole spectrum of -- objects. We apply a similar principle to iron metallurgy, given that iron and bronze metallurgy were in constant exchange in the context of cross-craft interaction. Indeed, it is possible to identify parallel examples, but they seem to be specific to a particular time and place. It seems that during the first few centuries after the adoption of iron in the ancient Aegean (11^th^ and 10^th^ centuries BCE), attempts to experiment were done on relatively large tools and implements ( > 15 cm), such as an adze from Sardis (cat. No. 127), which was produced by welding of steel and iron rather than by the means of conventional carburisation to create a product of a higher quality [53]. In contrast, small-scale objects ( ≤15 cm) such as the knives from Clazomenae and Asarlık were manufactured from wrought iron, and cold working was applied on the cutting edge in order to harden it [45]. On the other hand, in the Archaic period innovative approaches to iron working were apparently reserved for the production of smaller formats of specific function. Ü. Yalçın’s research at Miletus, for example, has demonstrated that more attention was paid to a nail-tip than to a massive spear head [3]. This observation is similarly echoed by the analysis of weapons and large implements from Mycenae [40] and Sardis [53]. Advanced technological processes visible in the UHCS microstructure were also deployed in the production of a small knife from Didyma [59]. The present study explores this phenomenon further with a larger assemblage of relatively small sized utilitarian iron objects from Didyma.

## 2. Materials and methods

### 2.1. Materials

More than 40 iron artefacts from Didyma were sampled within the framework of the broader analytical programme conducted by the authors. They all originate from clear stratigraphic contexts and have a direct or indirect relationship to production activities at the site. Out of these, eight objects fit the additional two key aspects of our methodological considerations – that they are small, utilitarian objects, most probably produced locally – making them a distinct subgroup for this study (Table 1). The remaining objects include 13 weapons and 22 implements. While the (predominantly large) weapons are dedications and do not seem to have been locally produced, the appraisal of knives and sickles is trickier, as they were manufactured in both small ( ≤ 15 cm) and large ( > 15 cm) formats and were used for different purposes in ritual context as part of ritual paraphernalia as well as commonplace implements in the sanctuary [21,63]. Therefore, they might, but they equally might not, meet the parameters outlined above and cannot be used to test the assumption of interplay between production, function, and context or practices of use. For example, a small knife from the Archaic deposit at Didyma, which has been previously analysed, is possibly of central Anatolian origin, and thus not a distinct local product representative of local technological milieu [59]. Understanding exact interrelations is therefore key in order to inform on the possibilities and limitations of the available technological knowledge.

**Table 1:** Analysed artefacts from Didyma. Contextual and analytical data on object marked with * have already been published in [59].

No.	Inventory number	Artefact class	Artefact type	Sector	Layer/ Level	Chronological scope
**1**	**MM03-277a**	Hammer	With a pointed peen	Taxiarchis hill	Post-Archaic limestone chip layer	Archaic to Hellenistic (700-300 BCE)
**2**	**MM01-103a**	Anvil (?)	‘Einsteckfäustel’	Taxiarchis hill	Ashy level	Second quarter of the 7^th^ to the first quarter of the 5^th^ century BCE
**3**	**04-BN12**	N.N.	Amorphous object	Temple area, Trench B, North sector	Layer 12	600-575 BCE
**4**	**MM09-271**	N.N.	Amorphous object	Taxiarchis hill	Ashy level	Second quarter of the 7^th^ to the first quarter of the 5^th^ century BCE
**5**	**MM03-280**	Anvil	Block	Taxiarchis hill	Reddish-brown loamy layer (part of earth embankment)	Second quarter of the 7^th^ to the early 5^th^ century BCE
**6**	**MM01-831**	Chisel	Cold-working	Taxiarchis hill	Ashy level	Second quarter of the 7^th^ to the first quarter 5^th^ century BCE
**7**	**MM01-581a**	Punch	Pointed	Taxiarchis hill	Ashy level	Second quarter of the 7^th^ to the first quarter of the 5^th^ century BCE
**8**	MM01-582*	Knife	With convex curved blade	Taxiarchis hill	Ashy level	Second quarter of the 7^th^ to the first quarter of the 5^th^ century BCE

**Fig 3:**
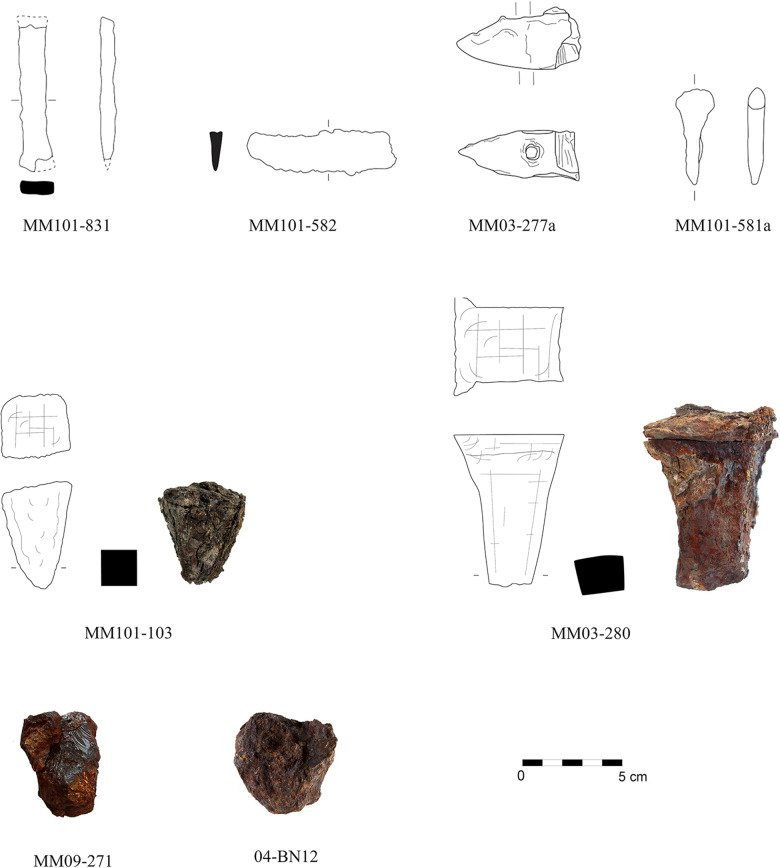
Analysed artefacts from the Sanctuary of Apollo in Didyma. Photo and drawing by M. Verčík; digitisation by M. Lelovič.

The bulk of the objects discussed in this article are tools for precision work used on non-ferrous metals, wood, bone, or soft stone (Fig 3): short and very light hammer MM03-277a with a pointed peen and a massive head with a square face suited for delicate forging; short punch MM01-581a with a massive point used in precious metallurgy; massive chisel MM01-831 with a solid square shaft and a tapered blade of triangular shape used in cold-work to cut metal sheets or for masonry working; and small block anvil MM03-280 with a relatively square head with a flat or convex face. The last object MM03-280 is unique; parallels are hardly known from ancient Aegean, and only La Tène parallels indicate its possible use in fine forging [7]. From a formal point of view, which is strongly affected by the state of preservation, this artefact has been described as anvil. However, the presence of a beard – deformed edges at the top section due to hammering – argues against its use as an anvil. A final interpretation of the form and function of the object must therefore remain open; until further comparative finds do not refute this, the description as anvil currently appears to be the most likely.

This group has been complemented by three corroded objects, which are assumed to be either tools, parts of tools, or semi-formed products. Object MM01-103a belongs to a group of small pyramidal objects with a tetragonal or square cross section with a flat face. A conclusive morphological identification is not possible due to the poor state of preservation, but the narrow pyramidal shape and square body resemble that of La Tène and Roman anvils of the ‘Einsteckfäustel’ type, which were originally embedded in a stone or wooden base by the means of a rod [64–66]. A description as cleaver or wedge applies only with some reservation, since the well-known ancient Greek masonry tool featured a wedge-shaped form [21]. The amorphous objects MM09-271 and 04-BN12 largely elude an adequate definition. However, their solid state, affected only little by corrosion inside, and semblance of a pyramidal shape suggests that they might have been parts of (broken?) tools or semi-formed products or billets rather than metallurgical waste; an object of similar form and structural characteristics from Archaic Miletus has previously been identified as part of a billet [3].

Block anvil MM03-280 and amorphous object 04-BN12 have been found in the Late Archaic levels predating the destruction layer of the early 5^th^ century BCE. Hammer MM03-277a was discovered in the post-Archaic limestone chip layer, which forms a massive sealing on the Taxiarchis hill. Metal objects are represented only sporadically when compared to the Archaic and Hellenistic pottery in this level, except for architectural fittings. The hammer could have thus belonged to the Archaic assemblage of tools and implements. In conclusion, the occurrence of the remaining five artefacts in the ashy layer is consistent with the interpretation of this deposit as a ‘rubbish’ dump after the Persian destruction, which devastated not only the Temple of Apollo but also all the cult and non-ritual infrastructure at the sanctuary.

### 2.2 Analytical method and sampling

A rotary tool equipped with air-cooled diamond discs was used to cut metallographic samples from all analysed objects. Due to official permissions and practical considerations, we were unable to cut full cross-sections of the objects. Instead, we adopted a strategic approach to sampling and selected sections that would provide the most information about the forming and heat treatment processes, which directly impacted the functionality of the objects. The sampling direction was chosen to allow observation of material properties at the working edges, as well as the differences between the surface and the core. For example, two samples were taken from anvil MM01-103a, one from the upper flat section and one from the lower pointed section. Similarly, both the flat and pointed ends of hammer MM03-277a and chisel MM01-831 were sampled. Punch MM01-581a was completely corroded, but its tip was sampled to analyse the metal remnants and ghost structures in Scanning Electron Microscope (SEM). Samples were mounted in epoxy and then ground on a rotary disc with SiC papers of 320, 600, and 1000 grit. Diamond suspensions of 6, 3 and 1 microns were used for polishing. The microstructures of the samples were revealed by using 1% Nital etchant, except in the case of a sample from punch MM01-581a whereby no solid metal was observed under light microscopy.

We recorded metallographic images of the samples digitally with Nikon E-Pol 200 optical microscope (OM) at varying magnifications. A Zeiss Sigma 500 SEM was used to identify structures that could not be seen clearly with a light microscope. In addition, chemical composition of bulk metals and slag inclusions was measured using an energy-dispersive X-ray spectroscopy (EDS) instrument attached to the SEM. Micro-hardness evaluation was made by employing the Vickers technique with an HV-1000Z model hardness tester from Pace Technologies. The tester’s accuracy was ascertained by measuring two standard samples (with hardness of 468 and 712 HV0.2) under 200-gram load, revealing a maximum error rate of 4%. The micro-indenter was placed on a minimum of five areas per sample that were free from corrosion and slag inclusions.

## 3. Results

### 3.1. Characterization of objects

The results of metallographic analysis and microhardness testing are summarised in Table 2 and can be described as follows.

**Table 2:** Summary of analytical (metallography and hardness measurement) results. Contextual and analytical data on object marked with * have already been published in [59].

No.	Inventory number	Artefact class	Micro-structure features	Hardness (HV0.2)	Materials
**1**	**MM03-277a**	Hammer	Equiaxed (slightly acicular) ferrite and coarse pearlite	140.9–149.9	Homogeneous medium carbon steel (0.3% - 0.4% C)
**2**	**MM01-103a**	Anvil (?)	Acicular ferrite, pearlite – normalised ferrite, pearlite	94–175.30	Heterogeneous – low to medium carbon steel between 0.05% C and 0.4% C
**3**	**MM03-280**	Anvil	Fine pearlite nodules, plate/lath martensite	300.6–682.7	High carbon steel (over 0.8%)
**4**	**MM09-271**	N.N.	Fine pearlite nodules, plate martensite – in a few locations hypoeutectic ledeburite	538.7–871.9	Ultra-high carbon steel (over 1.2% C) – over 2% C where ledeburite is present
**5**	**04-BN12**	N.N.	Fine pearlite nodules, plate/lath martensite – in the decarburisation zone: acicular ferrite, degenerated pearlite	113.5–779.5	High carbon steel (over 0.8%) – low carbon steel in the decarburisation zone (only a small region)
**6**	**MM01-831**	Chisel	Proeutectoid grain boundary cementite, cementite laths, fine pearlite	229–291.2	Ultra-high carbon steel (over 1.2% C)
**7**	**MM01-581a**	Punch	Proeutectoid grain boundary cementite	N/A	High carbon steel in the pointed end (over 0.8%)
**8**	MM01-582*	Knife	Proeutectoid grain boundary cementite, cementite laths, pearlite	N/A	Ultra-high carbon steel in the core (over 1.2% C)

**Fig 4:**
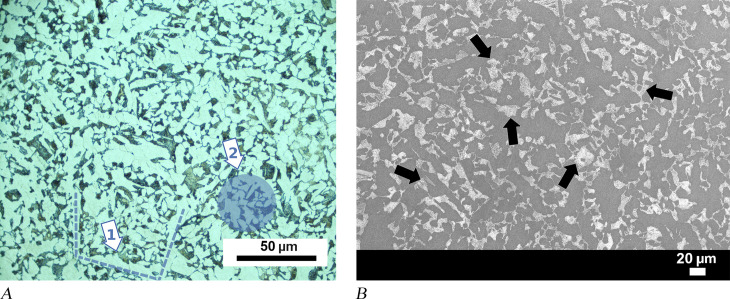
Analytical results for hammer MM03-277a. (A) Examples of phases detected in the object: (A1) proeutectoid grain boundary allotriomorphic ferrite formed along the prior austenite boundary, shown with dashed lines; (A2) the blue circle illustrates slightly acicular idiomorphic ferrites and pearlites. (B) An SEM image of the same sample shows pearlite grains visible as light-coloured patches (some examples of pearlite grains are indicated by black arrows).

Hammer MM03-277a was sampled at the flat and pointed ends of its metal head. Metallographic images of both samples show a homogeneous distribution of microstructures, including proeutectoid grain boundary ferrite, slightly acicular ferrite, and small pearlite grains (Fig 4A). The amount of pearlite and ferrite in the SEM images indicates a carbon content between 0.3% and 0.4%, which is consistent with a composition of medium carbon steel material (Fig 4B). The tool appears to have been made from highly refined metal, as slag inclusions are minimal. Hardness measurements indicate that the different points of the samples fluctuate within the range of +/−10 HV0.2, and the average hardness of both samples is 145 HV0.2. These observations confirm the homogeneity of the carbon amount in the flat pointed end sections of the tool.

After cleaning the carbonised wooden remains inside hammer MM03-277a, a narrow channel was revealed. Therefore, a wooden shaft must have been inserted in the hole inside the metal head. To create such a channel, a medium carbon steel material might have been chosen because of its relatively soft and ductile nature. The size and shape of the object indicate that it was intended for sensitive working, perhaps jewellery-making or decorating, involving flattening of soft metals with its flat side, embossing them with the pointed end, or by using it to strike a chisel to shape objects. For the purposes of such operations, the hardness of the hammer was not relevant at all, its weight would have sufficed.

**Fig 5:**
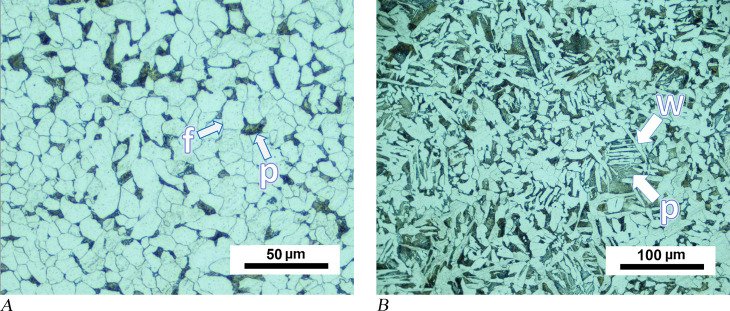
Analytical results for pyramidal-shaped object MM01-103a. (A) OM image of a sample from the upper section of the object shows equiaxed grains of ferrite (one grain is marked with a letter “f”) and pearlite (dark areas, one grain is labelled with a letter “p”). (B) Widmannstätten ferrite (an example marked with a letter “W”) and degenerated pearlite (a grain labelled with a letter “p”) were observed in another section of the same sample.

Microstructures of low carbon steel were observed in samples taken from the upper and bottom portions of pyramidal-shaped object MM01-103a. In most sections of the samples, the microstructure consisted of equiaxed ferrite grains and a minor amount of pearlite (Fig 5A). Microstructures indicative of higher carbon content were observed only in a small region of the sample in the upper part of the object. In this case, Widmannstätten ferrite and degenerated pearlite appear to be in a heterogeneous state (Fig 5B).

**Fig 6:**
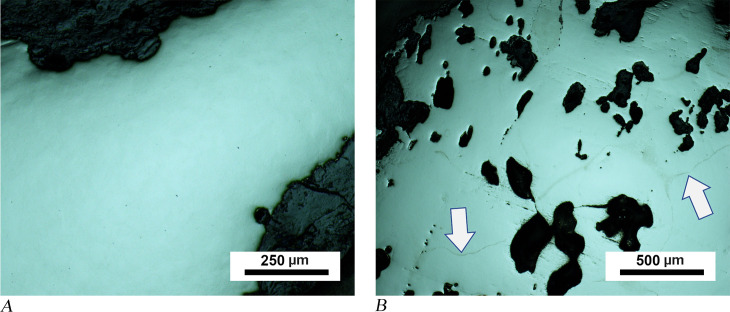
Analytical results for anvil MM03-280 and amorphous object MM09-271. (A) OM image of the MM03-280 showing clean metal without inclusions. (B) Dark corrosion areas and network of grain boundary lines (identified by two arrows) are shown in the image of unetched sample from amorphous piece MM09-271.

Material characteristics of anvil MM03-280 and amorphous objects MM09-271 and 04-BN12 are similar. A microscopy image of an unetched sample taken from an upper corner of anvil MM03-280 shows a clean metal area that is enclosed by a thick corrosion layer (Fig 6A). This suggests that the metal was highly refined and consolidated before shaping, as there were almost no slag inclusions visible. This observation contrasts with amorphous pieces MM09-271 and 04-BN12 in which several dark areas were observed in the bulk metal part of the unetched sample. Energy-dispersive X-ray spectroscopy (EDS) of the dark areas in MM09-271 revealed that their chemical composition corresponds to the oxide forms of iron, and only a few of them featured slag inclusions. Additionally, some grain boundary-like lines dividing the surface and forming polygonal shapes independent of the dark pores were observed in the optical microscopy images of the sample from the MM09-271 (Fig 6B).

**Fig 7:**
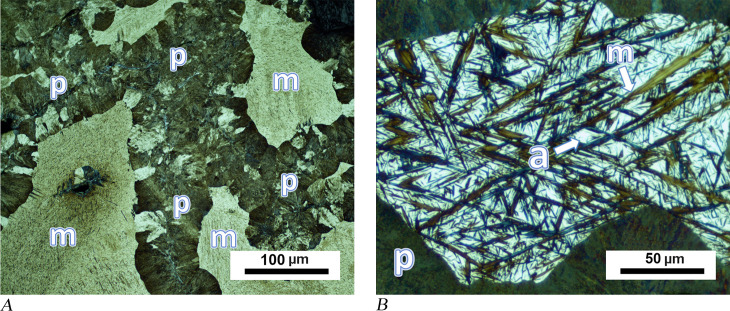
Analytical results for amorphous objects 04-BN12 and MM09-271. (A) Metallographic image of 04-BN12 shows pearlite colonies (dark areas marked with a “p”) and martensite (marked with an “m”). (B) In sample from MM09-271 (amorphous object), martensite plates (needle-like forms marked with an “m”) were observed in the austenite matrix (white background marked with an “a“) in between pearlite nodules (marked with a “p“).

Etching of samples from anvil MM03-280 and amorphous objects MM09-271 and 04-BN12 revealed similar microstructural features. The sample taken from the bottom part of 04BN-12 contains 60–70% pearlite colonies formed by the accumulation of nodules (seen as dark brown regions), and the rest is martensite (Fig 7A). Pearlite formed by a eutectoid reaction has a multi-layered morphology consisting of ferrite and cementite. However, Fig 7A shows that the layers of ferrite and cementite are so fine that it is not possible to distinguish the interspace between them through the light microscope. This fine lamellar structure of pearlite is the reason for its exceptional microhardness value, which was measured at over 500 HV0.2 in the pearlite zones (Table 2). In the same image, the surface boundaries of pearlite show a circular growth of nodules. The possible centres of these nodules follow prior austenite boundaries, similarly to those detected in the microscope image of unetched sample from MM09-271 (Fig 6B). Based on these observations, these lines visible in Fig 6B can be interpreted as prior austenite grain boundaries that acted as nucleation centres for the pearlite. Austenite, which is a high-temperature iron-carbon alloy phase, is stable above the eutectoid temperature of 727°C. Prior austenite grains appear to be coarse as some of the grains have been measured at 500 microns.

**Fig 8:**
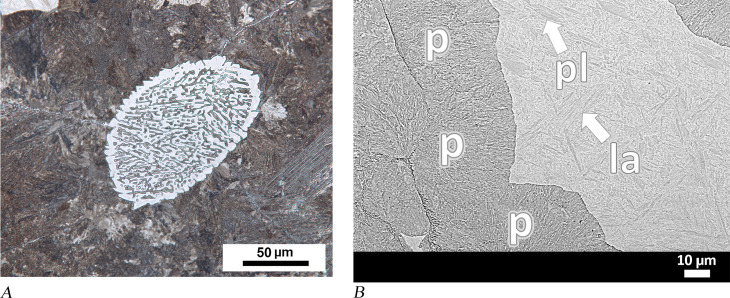
Analytical results for amorphous object MM09-271 and anvil MM03-280. (A) OM image of sample from MM09-271 shows eutectic ledeburite, an elliptical structure in the middle surrounded by a cementite rim with a fine pearlite background. Cementite is represented by the white areas, while fine pearlite can be seen in the dark areas between cementite. (B) Light grey area in the SEM image of MM03-280 represents a martensite portion, a mixture of plates (one plate is marked with a “pl”) and laths (a pack of laths is marked with a “la”).

The other microstructural component shown in Fig 7A, martensite, which has an acicular pattern in metallographic images, developed from a high-temperature phase (austenite) with rapid cooling, e.g., by quenching. Martensite is the hardest form of iron-carbon alloy; however, it is brittle. Microstructural analysis of the amorphous object (MM09-271) reveals martensite of the plate type with a characteristic feather-like structure (Fig 7B). Martensite plates are positioned on a white background matrix representing the retained austenite. The plate martensite only exists alone in compositions with above 1% carbon, while at lower carbon contents (between 0.6% and 1%) it occurs as a mixture with another type, lath martensite [67]. In addition to the plate martensite and pearlite nodules, hypoeutectic ledeburite structures have been detected at a few locations within amorphous object MM09-271. The eutectic constituent ledeburite has been formed as a result of the solidification of cementite and austenite. A rapid cooling process caused the austenite in this sample to form fine pearlite surrounded by cementite (Fig 8A). The presence of ledeburite implies that the composition at these locations corresponds to white cast iron, which is over 2% carbon, as well as the fact that the piece (at least at these locations) was liquidised during smelting. Due to the extreme fine lamellar spacing of pearlite and the plate type of martensite in object MM09-271, the carbon content of the entire sample can be estimated to be above 1%, which corresponds to UHCS containing 1.0 to 2.1 wt.% carbon. Even though pearlite nodules appear to be similar in samples from anvil MM03-280 and the other amorphous object 04-BN12, SEM revealed that the form of the martensite is different (Fig 8B). Martensite has been characterised in these samples as a mixture of plates and laths. Therefore, the steel used in the production of anvil MM03-280 and amorphous object 04-BN12 is also high carbon steel, most likely with a carbon content exceeding 0.8% (hypereutectoid).

The type and amount of martensite, the size of the austenite grains and the observations regarding pearlite formations provide a unique insight into the application of heat treatments on high carbon steel objects 04-BN12, MM03-280, and MM09-271. In contrast to anvil MM03-280, amorphous artefacts 04-BN12 and MM09-271 (perhaps semi-formed object or billets) were lightly forged as indicated by the low degree of consolidation and the undisturbed ledeburite structures in a few locations. All objects must have been heated above the temperature required for austenitisation temperatures (at minimum 850 – 950 °C, based on phase diagram for the suggested iron-carbon compositions), at which only austenite is stable, in order to obtain coarse grains of austenite visible in Fig 6B. Leaving the objects in smithing hearths at or above these temperatures – easily achieved by using charcoal and conventional airflow for an extended period – would result in coarse austenite grains. In practical sense, ancient blacksmiths would have observed the colour of the objects turning orange after the exposure to heat and waited to ensure that the centre of these thick objects was also heated. Next, they would have placed the items in a medium such as water or oil for rapid cooling in order to quench them.

Due to the distribution of martensite and nodular pearlite, it appears that a medium other than water was used in heat treatment. This may be due to the possibility of cracking when quenching these high carbon steel objects in water, as austenite is denser than martensite. Thus, the change in volume at the end of the transformation could cause internal stresses that would lead to faults; it is more likely for cracks to form in high carbon steel objects, particularly those of large volumes. Various quenching mediums (such as water and oil) result in varying cooling rates, leading to different microstructures. The final microstructure depends on many factors, such as the carbon content of the steel, quenching temperature, and the type of quenchant. As a generalisation, while the microstructure of steel turns to martensite fully after being quenched in water, a mixture of microstructural phases may be formed by a slower quenching in oil, as in the case of objects 04-BN12, MM03-280, and MM09-271. When an object is quenched in water, the cooling rate slows down through its core. The resulting microstructures can be similar to those of oil quenched steels around the core of thick objects. However, this is not the case for the objects from Didyma, as samples were taken from the corners and edges. Perhaps oil was chosen as quenchant to avoid cracks, and the blacksmiths might have been satisfied with the results. Due to the final microstructure consisting of brittle but hard martensite and more ductile pearlite, this heat treatment provided adequate mechanical properties, including impact strength, especially on the upper surface of anvil MM03-280, where some deformation due to hammering was observed.

Martensite types and hardness values reported in Table 2 indicate that quenching was the final heat treatment applied to object MM09-271, which exhibited martensite hardness exceeding 870 HV0.2. The value aligns well with the as-quenched hardness range documented for plain-carbon hypereutectoid steel [68]. Most commonly, quenching was followed by tempering, a process that involved holding the object at moderate temperatures in smithing hearths to mitigate brittleness. While the hardness values of martensite in objects MM03-280 and MM09-271 suggest a possible low-temperature tempering (around 200°C) based on Grange et al.’s observations [69], other factors influencing the final martensite hardness should also be considered. These include austenitizing temperature, self-tempering phenomena, and type of quenching medium (water versus oil).

**Fig 9:**
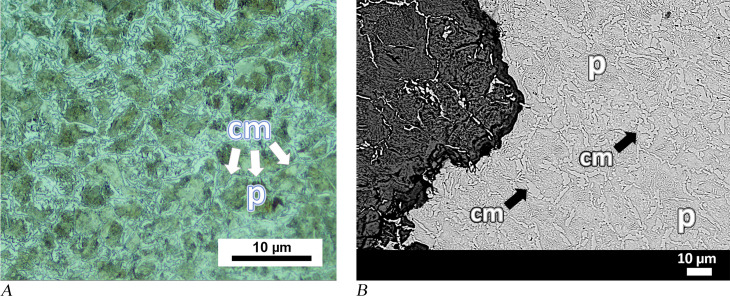
Analytical results for chisel MM01-831. (A) The proeutectoid (grain boundary) cementite network (cm) surrounds the pearlite grains (p) . (B) SEM image of the same sample shows some thickened grain boundary cementite, which took on a blocky shape, is indicated with black arrows and designated a “cm”. The corrosion area on the left of the image can be seen to contain metal remnants of the same type of cementite phase.

**Fig 10:**
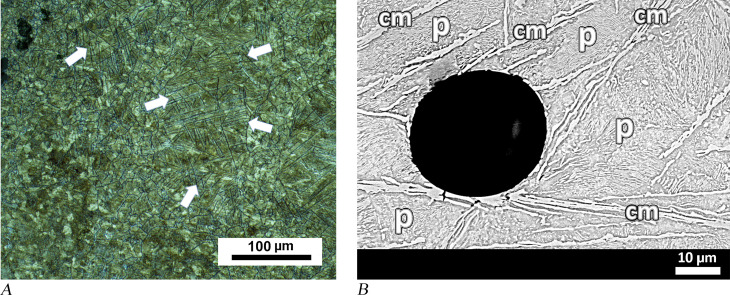
Further analytical results for chisel MM01-831. (A) Long acicular cementite plates (some are identified with white arrows) were observed in sample from the thin edge, in addition to grain boundary cementite. (B) SEM image of the same sample shows cementite plates (marked as “cm”) surrounding a circular pore. Pearlite lamellas can be seen in the background (marked with a “p”). Hardness values are between 229 and 291.2 HV0.2.

Analysing both edges of small chisel MM01-831 revealed microstructures indicating a hypereutectoid composition, corresponding with UHCS containing 1.2 to 2.1 wt.% carbon. A cementite network was found to border pearlite grains in the first sample taken from the thick end of the tool (Fig 9A). Cementite, a product of tough and brittle phase, is a compound of iron and carbon, Fe_3_C. As an element of pearlite, it may be associated with ferrite, but it can also be observed in high-carbon hypereutectoid steels as proeutectoid cementite. In addition to a continuous phase at the grain boundaries, proeutectoid cementite may also form side-plates (allotriomorphs) attached to the existing grain boundary network or separate needle-like (acicular) plates within the grains (intragranular). In Fig 9A, a grain boundary network of cementite phase is seen in the sample taken from the blunt end of chisel MM01-831. However, cementite plates were also observed in a few grains. SEM showed that most of the grain boundary cementite has become coarser and some has become blocky in shape (Fig 9B). A second sample from the same object, which comes from the thin working edge, exhibits proeutectoid cementite in the form of extremely long plates (Fig 10A). These plates are up to 100 microns in length and parallel to each other in coarse grains. During the cooling of the austenite phase, grain boundary cementite forms first, and at lower temperatures acicular allotriomorphs and intragranular cementite plates emerge. However, in the case of rapid cooling cementite forms such long intergranular plates. The hardness values of both samples taken from MM01-831 are between 229 and 291.2 HV0.2. This hardness would not be sufficient for working on materials such as hard stone.

The main drawback of UHCS is its extremely low ductility due to the hard and brittle cementite phases. Generally, the brittleness of the cementite is due to the grain boundary cementite network, which creates cracks under applied force and acts as a propagation path for cracks. Acicular cementite plates can also cause cracks due to their anisotropic character, but the cracks propagate in a more complicated manner compared to network cementite. The thin working edge of chisel MM01-831 is more unlikely to break during use, so observing acicular forms more clearly in this part raises the question if this section of the tool was deliberately produced in this way. A closer look at the microstructure reveals that the pearlite grains keep their lamellar form, and the plates of cementite can be considered as relatively undisturbed (Fig 10B). It therefore follows that the chisel must have undergone a final heat treatment, which most likely consisted of heating the object’s thin edge in the smithing hearth, which caused a full austenitisation (over 900°C), followed by a rapid cooling process. Microstructural composition suggests that air cooling rather than quenching would have been more fitting of the pattern observed. Even though this may not have been an optimal final heat treatment for a UHCS tool, it may have been beneficial in releasing the stresses generated during the shaping process.

**Fig 11:**
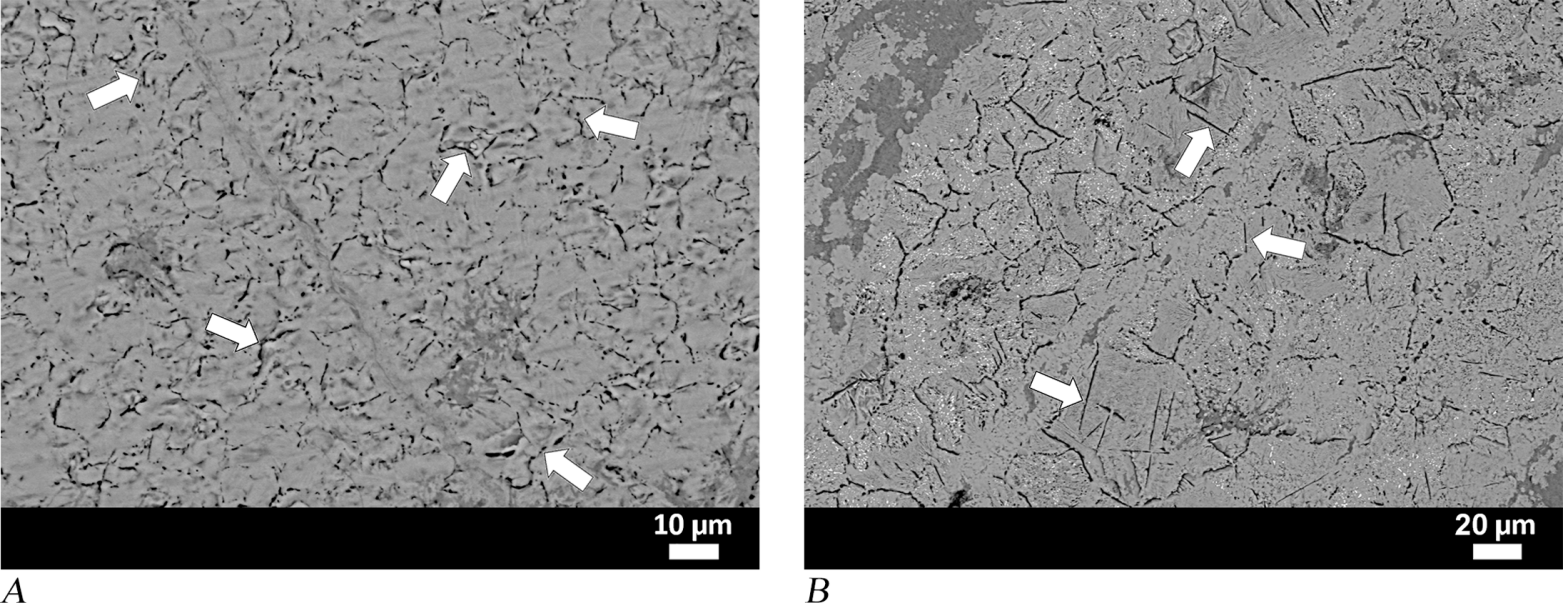
Analytical results for punch MM01-581a and knife MM01-582. (A) Sample from MM01-581a shows that ghost structures are associated with the cementite network and are visible as dark grain borders. (B) In sample from MM01-582, ghosts of grain boundary cementite and proeutectoid acicular cementite plates are also evident within the grains [59].

The last object, punch MM01-581a, was completely corroded. A few dark-coloured ghost structures were observed in the SEM image of the sample taken from the pointed end. Ghost structures refer to corrosion products that replace the original structure, which was consumed [59,60], and ghosts of carbon-rich microstructures at the grain boundaries can be recognised with their contrasting dark-coloured appearance [70]. Accordingly, the black-coloured network shown in Fig 11A likely represents the grain boundary cementite network. This phase implies that the pointed end of the punch was formed from hypereutectoid steel. Due to the limited areas in which ghost structures were detected in the sample, it was not possible to identify the full material characteristics and heat treatments applied to the tool.

## 4. Discussion

The results confirm the possibility that at Didyma small sized iron tools can be considered a specific ‘testing category’ of objects for experimentation undertaken by blacksmiths during the Archaic period. The analysed objects have distinct material features when compared to other iron-steel artefacts (i.e., weapons, implements, architectural fittings) of both small and large formats, all of which were investigated as part of the broader archaeometallurgical study in Didyma. Most of the finds presented are of high-quality iron, with a very small amount of slag inclusions in the uncorroded sections of the samples, rather than of average quality wrought iron, characterised by heterogeneous carbon content and slag inclusions in large quantities. Moreover, by taking samples from different sections of the objects we observed that each sample from the same object exhibited similar microstructures, indicating the homogeneity of the materials. In addition to this, the bulk of the finds was produced from hypereutectoid steel with the exception of pyramidal-shaped tool MM01-103a and hammer MM03-277a. Furthermore, the microstructures of possible semi-formed object/billet MM09-271 and chisel MM01-831 also demonstrate that their material is UHCS.

Punch MM01-581a was studied with an analytical method previously applied to corroded Archaic knife MM01-582 from the same site [59]. Both punch MM01-831 and corroded knife MM01-582 contained ghost structures of proeutectoid cementite. Moreover, acicular cementite plates and the thickness of the cementite, as observed in the centre of the knife’s cross-section, were interpreted as evidence for UHCS characteristics of the material (Fig 11B). In addition, our analysis demonstrated that both anvil MM03-280 and amorphous object or tool 04-BN12 are homogeneous steels with hypereutectoid composition. Detecting such a high-quality homogeneous high carbon steel as the material of these objects raises several questions concerning the mode of production, forming, and motivations behind the choice of material in terms of the object’s function, as well as the context of its use, i.e., the socio-cultural context of the available technology. These key aspects therefore need to be considered in order to contextualise the emergence of UHCS in the Archaic period.

### 4.1. Modes of production

There are different ways in which the production of UHCS objects could have been achieved. In antiquity, a bloomery furnace served as the primary means of smelting iron ores. During this so-called direct production process, charcoal was burned as a source of heat and carbon to provide the required temperature and reducing atmosphere for the reduction of ores to metal. In the narrow section of the furnace, iron oxide minerals converted into metallic iron grains, which agglomerated in the lower parts of the furnace and created the final product called bloom. For the successful implementation of this process, gangue minerals in the ore, semi-reduced iron oxides and furnace materials that reacted during the process formed slag (a mixture of iron silicates with fluxes), if any, were added. Bloomery iron is the product of such smelting process. Archaeological evidence, thermodynamic evaluations and experimental studies have all shown that the notion that direct reduction process can only produce low, soft carbon iron is not valid [71]. Adjustments made to various variables, such as fuel and ore feeding regime, type of ore, presence of other oxides in the ore, such as manganese oxides, the flow rate and position of air supply, furnace design, etc., could result in the production of every type of iron-carbon alloy from carbon-free iron to pig iron.

In general, ancient smelters must have sought to produce blooms with low carbon content, as developed skills and experience in smelting were needed to achieve a homogeneous carbon-rich steel suitable for working in blacksmiths’ hearths. Yet in some sections of an ordinary bloom, one may observe localised regions that were highly carburised, such as the crown material [72]. During the refinement process, these limited sections were either removed from the bloom or consolidated with other sections, resulting in a heterogeneous matrix [73].

Whenever the smelting conditions favoured the production of cast iron, the available technological knowledge of the time was insufficient to process the product further. In archaeological contexts, evidence of this lack of knowledge can be found in the form of discarded metal masses, such as for example at Sirkeli Höyük in Cilicia [74].

An existence of deliberate cast iron production in the Aegean has been based on the written evidence only. According to Aristotle’s description of steel production in Meteorologica (383^a^32), wrought iron was first melted to remove slag content, and then resolidified. By repeating this process, iron should have become cleaner, more homogeneous, thus turning into steel. Scientists have discussed this procedure and proposed that it explains the refinement of a bloom by heating and forging [43]. E. Photos [75] has suggested a connection between Aristoteles’ steel-making definition and the depictions of a cauldron positioned atop a metallurgical furnace on Greek vases from the 6^th^-5^th^ centuries BCE [17]. According to her, these cauldron-bearing furnaces were designed to produce steel for specific purposes, namely for weapons and armour, by the process of decarburising cast iron [75]. The difficulty in determining whether the finds from Didyma may be the products of such a process lies in the fact that refined iron and bloomery iron have similar metallographic features [76]. A tradition of indirect steel production would, however, leave traces, such as easily recognisable cast iron remains. Yet to our knowledge there are no iron objects or metallurgical remains from the ancient Aegean or western Anatolia that provide evidence for such a processing of cast iron.

High carbon steel can also be produced by carburising wrought iron. In this case, iron is kept at high temperatures in a reductive atmosphere rich in carbon for a sufficiently long period of time. Such a setting can be achieved inside a smithing hearth by covering the object with charcoal, or in a more controlled manner, by heating it in a sealed ceramic pot containing iron and carbonaceous materials. Conophagos and Papadimitriou [77] have suggested that the depictions on the previously discussed Greek vases must demonstrate an iron-carburising system used to obtain case-hardened iron sheets. To support this, the authors studied clamps from the 5^th^ century BCE Erechtheion in Athens metallographically. The clamps from the temple were produced through forge welding of layers of medium-carbon steel and iron. If the carburisation process was intense enough, it would have produced steel with high carbon composition. Consequently, the authors reported that the carbon content created by this process was as high as 1% in some layers [77]. In thick objects such as the finds from Didyma, it is, however, not possible to obtain a homogeneous carbon content using a diffusion-controlled process. Application of case hardening, whether in a smithing hearth or a ceramic pot, would have led to carbon enrichment at the surface, resulting in a noticeable degradation of microstructure toward the object’s center. If carbon diffusion takes place in liquid state of steel, which requires temperatures over 1400°C, homogeneous high carbon steel can be produced. These products are known as crucible steels, which are slag inclusion-free and mostly hypereutectoid, and are the result of heating iron and carboniferous material at extremely high temperatures in enclosed ceramic containers. Although there is material evidence of crucible steel production and use in Tamil Nadu, India in the mid-first millennium BCE [78,79], the earliest centre producing crucible steel ever found in Anatolia dates to the 13^th^ century CE [80].

Our analysis suggests that the homogeneous hypereutectoid steel detected in tools and semi-formed objects from Didyma was either completely or partially liquefied during the smelting process, but we recognise that similar conditions could have been created through crucible steel production process. The presence of ledeburite phases in amorphous object MM09-271 (seen on Fig 8A) and spherical gas holes in chisel MM01-831 (visible in Fig 9B) are consistent with these two scenarios. Studies of discarded metal lumps, semi-formed bipyramidal iron bars and finished tools such as steel files found at Magdelensberg, the manufacturing centre of Ferrum Noricum during the Roman period, revealed the manufacture of hypereutectoid steel in one-step smelting process [81]. During experimental studies, it has been established that with certain adjustments, the ore that comes into direct contact with unburned coal pieces in the throat section of the smelting furnace is transformed into molten cast iron under the influence of CO-rich atmosphere and high temperatures, with molten cast iron then flowing down into the lower regions of the furnace. Due to decarburisation in the oxidation zone under the influence of air blown through the tuyeres, the carbon content of the metal mass decreases to a steel to hypereutectoid steel composition. Even though manganese content in the ore used for Ferrum Noricum is considered to have contributed to the high carburisation rate and protection against decarburisation, similar results can be achieved through smelting manganese-poor ores in underground furnaces [43]. In the *Naturalis Historia* (34.41), Pliny the Elder mentioned smelting techniques for iron ores, some of which could yield high-quality, hardenable steel suitable for crafting anvils. This is noteworthy as archaeometallurgical studies conducted in the ancient Milesia on Archaic period material demonstrate early traces of similar metallurgical innovations: in Didyma this is demonstrated by anvil MM03-280 with high-carbon composition, while in Miletus [3] by the pyramidal-shaped ‘billet’ with ferritic to ledeburitic microstructural composition.

Archaeometric studies of iron artifacts from the archaic Greek mainland do not indicate the presence of hypereutectoid steel. A study of Classical objects from the Acropolis in Athens observed only a lower carbon content and a more homogeneous ferritic matrix on three double-T clamps from the Parthenon [35], which contrasts with the clamps from the Erechtheion mentioned earlier, which were formed of forge-welded layers of medium-carbon steel and iron [38]. The 5^th^ or 4^th^ century BCE iron shells from wooden poles of an ancient pier near the city of Amphipolis were forged from hypoeutectoid steel and thus illustrate that the use of high-quality steel for architectural fittings was not consistent at that time [41]. Moreover, Rostoker and Gebhard [33] have concluded that low carbon bloomery iron was employed to produce iron tools and weapons in the Sanctuary of Poseidon at Isthmia during the Hellenistic period. Finally, a metallographic analysis of knives and swords from the Early Iron Age cemetery at Vergina has attributed a higher carbon content at the cutting edges rather than at the core of artefacts to preferential carburisation [34]. Even so, the highest amount of carbon in the carburised locations barely exceeded medium carbon composition.

Moreover, the presence of hypereutectoid steel objects has rarely been noted in archaeological excavations even beyond the ancient Aegean. One of the earliest examples is an Early Iron Age bracelet (1200-1050 BCE) recovered together with iron jewellery in Jordan’s Baq’ah Valley [82]. Some bracelets and rings had a homogeneous eutectoid carbon content, but an analysis of one of the bracelets showed coarse cementite globules throughout the sample. Considering the function of the object as jewellery, it prompts the question of what motivated the craftspeople to deliberately manufacture UHCS. Another early example of UHCS objects is a single-edged knife dated to the 11^th^ century BCE, from Idalion on Cyprus. It was recovered as a fully corroded blade fragment, but microstructural characteristic of UHCS were detected in a sample taken near the broken end, suggesting that the blade had been quenched but not tempered [83]. However, no recognisable traces of martensite were visible in the published microscopic images of the corroded sections. In the southern Levant, a UHCS object dating to the 7^th^ century BCE was found in Hazor. Metallography of this square-sectioned tool showed acicular cementite plates similar to the microstructural constituents of chisel MM01-831 and blade MM01-582 from Didyma [84]. A UHCS tool found at a Late Roman-Iron Age site in Heeten, the eastern Netherlands, was classified as a punch [1]. While the tip of this punch was hardened by quenching, the back part was left to cool slowly. Depending on the applied heat treatments, various structures such as spheroidised cementite, proeutectoid cementite network, and intragranular acicular cementite were detected in different sections of the object. These types of structures have been frequently encountered in the microstructural compositions of crucible steel ingots and tools from the Medieval Ages [80,85,86]. In sum, considering the scarcity of early and contemporary examples, the Archaic hypereutectoid steel tool assemblage recovered in Didyma (and Miletus) is unique in terms of deliberate production and heat-treatment process.

### 4.2. Modes of use and functionality

Material properties and performance characteristics of objects made of UHCS provide clue to their function and use. The premise here is that desired functionality is not based on inherent properties of metal or the alloying process, but on the additional thermal and mechanical treatment that produce beneficial effects such as hardness.

The amorphous artefacts of square or rectangular appearance MM09-271 and 04-BN12 are likely semi-formed iron objects or billets. Fracture lines observed on the surface of MM09-271 suggest that the object may have been broken during forging and was therefore discarded. Localised brittle ledeburite structures (visible in Fig 8A) explain why fracturing occurred during hammering. Since high carbon steel tools such as chisel MM01-831 and knife MM01-582, in addition to an unpublished a tool and a semi-formed bar from Miletus (no. Z-04-21-8 and no. Z-03-29-12), demonstrate that such materials were successfully forged into tools. Therefore, there is no reason to conclude that all high carbon iron bars were discarded this way.

Our examination of iron bars from Didyma indicates that the workshops there were stocked with materials of diverse characteristics. Our study shows that the samples from the semi-formed iron smithing material consisted of high carbon areas, in addition to high quality steel blocks with homogeneous eutectoid composition as well as those with heterogeneous low carbon compositions. A hardening process was applied only to high carbon iron bars (e.g., an object from Miletus, no. Z-03-29-12), as evidenced by hardness values approaching 900 HV. As these products would have been shaped further, the application of quenching in the first place would not have been justified. Yet the high hardness of these iron bars, a property that could have been tested by ancient blacksmiths, may have been a factor in their decision to import.

Anvil MM03-280 has a micro-structure of high carbon steel, and unlike the other two high-carbon semi-formed objects or bars MM09-271 and 04-BN12, it was produced from a well-consolidated metal. It is worth noting that both pearlite colonies and martensite were discovered in the samples taken close to the surface of all three objects. These microstructural constituents were formed by a specific cooling process, most likely oil quenching. It thus appears that anvil MM03-280 and the two amorphous objects MM09-271 and 04-BN12 might present the earliest archaeological evidence of using oil for quenching. As the literary evidence provided by Pliny the Elder (Hist. Nat. 34.41) suggests, oil and various types of water were used for quenching in the Roman world to produce high-quality steel. Oil-quenching was used for small tools, as he specifies shortly after discussing the production of hardened steel for anvils. The idea of using oil in quenching was likely experimented with to circumvent possible oxidizing conditions that could lead to decarburisation on surfaces of hot objects that water could create when used as a quenching medium. Therefore, oil would have been a more suitable alternative for preserving high carbon content. Additionally, oil-quenching would have likely imparted excellent mechanical properties, namely high hardness and moderate wear resistance while maintaining toughness through shallow hardening near the surface. Cooling rate could be expected to be slower at the core of these objects, leading to the formation of a fully pearlitic structure. The softer core of the tool would have been protected from impact fractures caused by hammering, a vital feature for small-sized anvils like MM03-280. This combination of properties was particularly critical given the limited size of their working face. Without such a measure, they could have swiftly incurred damage.

Determining the rationale behind selecting UHCS steel for chisel MM01-831 is more complicated. It is obvious that this was not an accidental production as we have identified another UHCS chisel at Miletus, which has not yet been published (no. Z-04-21-8). Cementite, both in the form of grain boundary networks as well as in the form of intragranular laths, is brittle. Using UHCS chisel on hard materials such as iron, bronze, and marble may be problematic because of its brittleness and poor toughness. The chisel was, therefore, more probably used on softer materials (such as soft-stone, wood and bone), or soft metals used for cutting metal sheets, wire, and casting surpluses [16,87,88]. After all, a heat treatment might have been applied to the edge of the tool to resolve this issue. In contrast, punch MM01-581a was most likely used for engraving bronze vessels or decorative plates, in addition to chasing and hallmarking. This is all the more important as this technique could not have fully been applied before the wide use of steel due to the mechanical properties of the tools used [87,88].

### 4.3. The context of production and consumption

Except for two semi-formed artefacts, the analysed finds represent a specific category of tools, which were exclusively used for precision work on bone, wood, soft stone, and non-ferrous metals. While the craftspeople who work with iron, bronze, and lead might sometimes overlap in terms of skill, as both archaeological and literary evidence suggest, there appear to have been genuine specialists working on precious and fine copper-alloy metallurgy in the ancient Aegean [24,89]. However, excavated workshops of gold- and silversmiths are still few in numbers: metallurgical remains, semi-formed objects, and ingots are known from the late Geometric phase of the Sanctuary of Apollo in Eretria [90], Late Archaic levels at the settlement of Thasos [91], and a 5^th^ century BCE workshop of Phidias in Olympia [92]. Objects without any stratified context include numerous stone moulds, and individual tools unearthed in the sanctuaries of Ephesus and Miletus [93]. The tool assemblage from Didyma adds to this evidence yet at the same time displays a broader array, although it does not comprise a complete ‘tool kit’ of gold, silver, and fine coppersmiths’ workshop(s) that operated within the sanctuary. The craftspeople were active in Didyma already at the beginning of the 6^th^ century BCE as evidenced by the anvil MM03-280 and semi-formed object 04-BN12 unearthed in a loamy earth embankment level on the Taxiarchis hill and in the Temple area NO. Based on the stratigraphic record of the chronologically later ashy level on the Taxiarchis hill, which includes cupriferous slags and spoiled casting [7], there was a specialised metallurgical activity in the sanctuary on the eve of the Persian incursion in the early 5^th^ century BCE. Whether it was a temporary or a stationary production cannot be established, as there are no traces of any fixed installations.

Strictly speaking, no decisive evidence has come to light that would enable us to directly associate the analysed tools with blacksmithing in Didyma during the Archaic period. The iron working in the sanctuary of Apollo is attested by the finds of SHB/PCB slags, ingots, and semi-formed objects [7], of which the majority revealed heterogeneous nature consisting of iron and low-carbon steel. Only the two semi-formed objects or bars (MM09-271; 04-BN12) form an exception.

The variation in the material features of the analysed assemblage indicates that similar production techniques were not commonly applied in a linear fashion. While anvil MM03-280, punch MM01-581a, and chisel MM01-831 were produced from hypereutectoid steel, small pyramidal object MM01-103a and hammer MM03-277a display heterogeneous or homogeneous low to medium carbon-steel. In addition to the tool assemblage, in our previous study we discovered ghost structures of proeutectoid cementite and acicular cementite plates in a sample from corroded UHCS knife MM01-582 from the Taxiarchis hill in Didyma [59]. This knife differs from other knives examined from the same site, which represent the average quality of wrought iron objects. On the one hand, the variation in the microstructural features and the materials used could refer to the different function of the analysed objects, if, for example, anvil MM01-103a was used for working materials other than those produced from UHCS steel. On the other hand, keeping in mind that there are no or very little morphological differences within the group of anvils, which could point to a different practice, an alternative interpretation is possible. The tools and the knife could be produced by different workshops or specialised craftspeople – e.g., those working on precision tools specifically – according to the requirements of the consumers.

Tools and working implements have frequently been interpreted by the scholarship as ‘personal’ artefacts inherently connected with craftspeople in antiquity [94,95], who are thought to have selected and maintained specific instruments until the decision was made to replace them. The archaeological evidence from the Archaic period attests the presence of such objects, alongside semi-formed objects and bars, deposited as offerings in the foundations of the Archaic temple at Kalapodi [96], in deposits of the 8^th^ to 7^th^ century BCE at the Temple of Hera Limenia in Perachora [97], or placed in a grave of ‘craftspeople’ in the Archaic necropolis at Panormos by ancient Didyma [98].

Could these UHCS tools be such personal artefacts produced or provided for the craftspeople (metalworkers, but also ivory- and woodworkers, or stonemasons) at Didyma? A review of Archaic iron workshop remains from Clazomenae, modern-day Urla, in northern Ionia [99], suggests that iron tools were discovered alongside numerous slags and bloom fragments (in a channel filled during the final phase of use of the Clazomenaean workshops). These were indeed attributed to the metalworkers. The analysis of bloom fragments from Clazomenae, however, showed that they have a heterogeneous content. In addition to iron and low-carbon steel regions, small areas around eutectoid composition (0.8 wt.% C) were detected in a sample from the cross-section of the bloom [55]. A similar type of heterogeneous bloom was recovered from another Archaic period smithing workshop at Kalabaktepe in Miletus [3]. Along with pieces of the bloom, several objects were analysed, including a nail no. TR-3/23, the tip of which revealed cementite needles and grain boundary cementite, which are comparable to the UHCS material found in Didyma. Our ongoing study of the Milesian iron objects confirm some use of UHCS for the production of tools, in addition to the presence of UHCS semi-formed object or bar.

This observation is not so surprising when considering other crafts in the region of the Milesia. In recent years, intensive analytical program targeting ceramic pots, as well as building and technical ceramics has shed new light on the possible links between Miletus and Didyma during the Archaic period [8]. Indeed, one cannot imagine the emergence of monumental architecture in Didyma during the 6^th^ century BCE without the rapid development of masonry techniques, documented at Miletus [100]. It can be thus postulated that tools for precision craftsmanship from Didyma were probably produced within the region of Milesia, and that the technological knowledge of UHCS was systematically used here.

Of course, we cannot currently rule out with certainty that these objects could have been produced elsewhere [101], Miletus was one of major economic and cultural powerhouses in the ancient Greek world during the Archaic period [102], described as the ‘Ornament of Ionia’ by Herodotus (5.28). It actively took part in mobility across the Mediterranean and Black Seas and maintained relations with Egypt and Lydia. The city’s success and networks must have stimulated prolific exchange and transfer of knowledge and technology. There are a few indicators for the activity of foreign craftspeople and metalworkers, such as similarities in ornamental decoration and moulds for jewellery at the site [103–105], but so far there is no evidence that this could have been the case for iron technology as well.

## 5. Conclusions

Metallographic and chemical analyses of a stratigraphically secured assemblage of iron objects from the Sanctuary of Apollo at Didyma dating to the Archaic period showed that it represents a unique assemblage in the Archaic Aegean. Specifically, the combination of analytical methods identified that the microstructure and material features of the objects represent the earliest evidence for a deliberate creation of UHCS in the ancient Mediterranean. Such homogeneous and slag inclusion free UHCS could have been produced in two ways. First possibility is that the tools were made from either partially liquefied bloom parts from the smelting process. The second is that they came from completely liquefied steel produced by a process similar to crucible steel production. Function of these objects as precision tools provides a good motivation for experimentation with UHCS, as their production and use happened in the particular setting of workshops attached to a major sanctuary. Variation in the production of these objects from Didyma and similar objects from nearby Miletus suggest variable knowledge of craftspeople, as evidenced by the only other UHCS objects from the Archaic period Kalabaktepe at Miletus, located only 8km from Didyma. This geospatial link points to interconnectedness and mobility of craftspeople within the Milesian peninsula.
